# The effect of different ferrule heights and crown-to-root ratios on fracture resistance of endodontically-treated mandibular premolars restored with fiber post or cast metal post system: an in vitro study

**DOI:** 10.1186/s12903-023-03053-4

**Published:** 2023-06-03

**Authors:** Qingfei Meng, Yuxin Chen, Ke Ni, Yingmei Li, Xinran Li, Jian Meng, Lijuan Chen, May Lei Mei

**Affiliations:** 1 Department of Stomatology, Xuzhou Central Hospital, Xuzhou, China; 2 Department of Stomatology, Xuzhou Clinical School of Xuzhou Medical University, South Jiefang Road No. 199, 221000 Xuzhou, China; 3 College of Stomatology, Bengbu Medical College, Bengbu, China; 4 Department of Stomatology, Xuzhou first People’s Hospital, Daxue Road No. 269, 221000 Xuzhou, China; 5 Faculty of Dentistry, University of Otago, Dunedin, New Zealand

**Keywords:** Crown-to-root ratio, Ferrule, Residual root, Fracture resistance, Post and core system

## Abstract

**Background:**

This study aimed to investigate the effects of different ferrule heights and crown-to-root ratios on the fracture resistance of endodontically-treated premolars restored with fiber post or cast metal post system.

**Methods:**

Eighty extracted human mandibular first premolars with single root canal were treated endodontically and cut from 2.0 mm above the buccal cemento-enamel junction, to create horizontal residual roots. The roots were randomly divided into two groups. The roots in group FP were restored with a fiber post-and-core system, while the roots in group MP were restored with a cast metal post-and-core system. Each group was divided into five subgroups with different ferrule heights (0: no ferrule; 1: 1.0 mm ferrule; 2: 2.0 mm ferrule; 3: 3.0 mm ferrule; 4: 4.0 mm ferrule). All specimens were subsequently restored with metal crowns and embedded in acrylic resin blocks. The crown-to-root ratios of the specimens were controlled at approximately 0.6, 0.8, 0.9, 1.1, and 1.3 of the five subgroups, respectively. Fracture strengths and fracture patterns of the specimens were tested and recorded by a universal mechanical machine.

**Results:**

Mean fracture strengths (mean ± standard deviation (kN)) of FP/0 to FP/4 and MP/0 to MP/4 were: 0.54 ± 0.09, 1.03 ± 0.11, 1.06 ± 0.17, 0.85 ± 0.11; 0.57 ± 0.10, 0.55 ± 0.09, 0.88 ± 0.13, 1.08 ± 0.17, 1.05 ± 0.18 and 0.49 ± 0.09, respectively. Two-way ANOVA revealed significant effects of different ferrule heights and crown-to-root ratios on the fracture resistance (P < 0.001), but no difference in fracture resistance between two post-and-core systems (P = 0.973). The highest fracture strengths of the specimen were found with the ferrule length of 1.92 mm in group FP and 2.07 mm in group MP, the crown-to-root ratio of which in 0.90 and 0.92 respectively., there is a significant difference in fracture patterns among the groups(P < 0.05).

**Conclusions:**

When a certain height of ferrule is prepared and a cast metal or fiber post-and-core system is restored for the residual root, the clinical crown-to-root ratio of the tooth after restoration should be kept within 0.90 to 0.92, so as to improve the fracture resistance of endodontically-treated mandibular first premolars.

## Background

The trauma or dental caries may lead to a large amount of tooth defect, which directly affects the chewing and aesthetic functions of patients. Placement of a post-and-core buildup has been recommended to restore endodontically-treated tooth with insufficient coronal structure [1–4]. The prognosis of the post-and-core restoration of the tooth can be affected by multiple factors, such as post material [5–9], ferrule design [8, 10–14], crown-to-root ratio (CRR) [14, 15], residual dental tissue amount [9, 16, 17] and adhesive system [18, 19].

It is generally accepted that more than 1.5 mm ferrule lengths can improve the fracture resistance of the tooth [12–14]. If the cervical level of the residual root is equal-gingival or subgingival, the surgical crown lengthening or orthodontic forced eruption may needed to obtain enough supragingival tooth tissue in favour of ferrule design, which may increase the clinical CRR of the tooth after full crown restoration. When CRR exceeds 1:1, the fracture resistance of the tooth may decrease [20].

However, there is little research on how to match the ferrule height with CRR to improve tooth resistance during restorative procedure. Hence, this study aimed to compare the effects of different ferrule heights and CRRs on the fracture resistance of endodontically-treated mandibular premolars.

Two post-and-core systems were used to restore the tooth. The null hypotheses were that the effect of different ferrule height (or CRR) or post material had no significant differences on the fracture resistance of endodontically-treated teeth, and there was no internal correlation between the ferrule height and CRR on the effect of the fracture resistance of the teeth.

## Methods

### Specimen preparation

Eighty sound human mandibular first premolars extracted for orthodontic reasons from patients aged 18–30 years who lived in the same locality were collected and used in this study. Written informed consent was obtained under a protocol approved by the Ethics Committee of Xuzhou Central Hospital (No.XZXY-LK-20220617-053). All the teeth were examined stereoscopically at 10× magnification with no cracks, or caries and stored in 0.9% saline solution at 4^o^C for no longer than 2 weeks [20]. The crowns were cut transversely 2.0 mm occlusally beyond the buccal cemento-enamel junction (CEJ) by diamond disks, and the residual roots with an average length of 15.0 ± 1.0 mm were retained. Standardized root canal preparations were completed with size 4 Gates-Glidden drills (Dentsply-Maillefer, Ballaigues, Switzerland) before cold laterally-condensed gutta percha points (Dentsply International Inc.,York,PA,USA) were placed to obturate the canals. After sealing the root canal orifice and root tip with paraffin, the roots were immersed in 0.9% saline solution for 1 week.

**Fig. 1:**
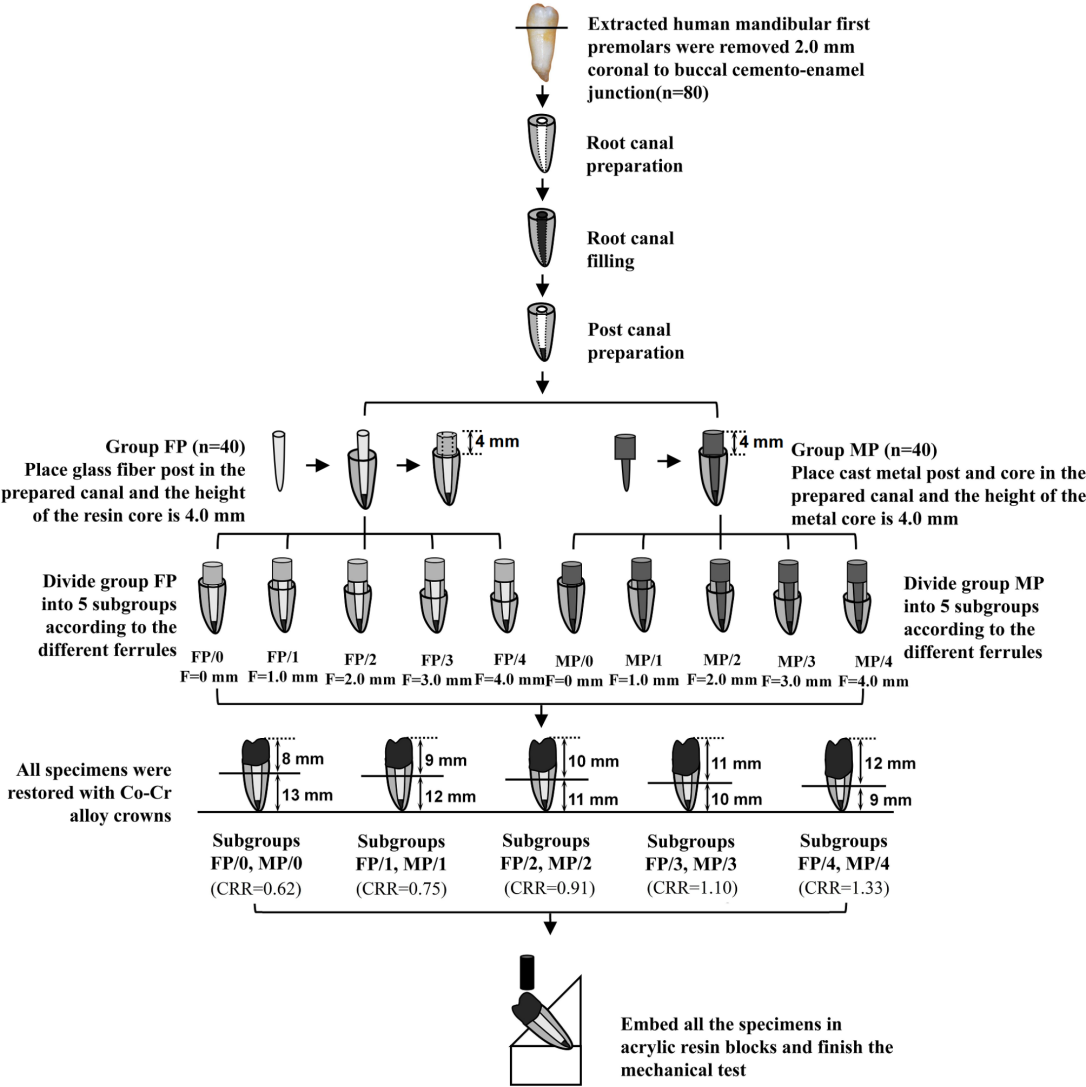
Schematic representation of the study process

The flowchart of the study design is shown in Fig. 1. A power calculation was performed to estimate the sample size using an effect size of 0.3, statistical power of 0.8, to determine a minimum sample size of 8 in each subgroup. Therefore, eighty specimens were prepared in this study. Eighty residual roots were equally divided into FP and MP groups according to a table of random numbers. Each group was assigned randomly into five subgroups with the height of the ferrule from 0.0 to 4.0 mm, which were recorded as FP/0 ~ FP/4 subgroups and MP/0 ~ MP/4 subgroups respectively. The root dimensions including the root lengths, the cross-sectional widths of the canal walls at the mesial, buccal, distal and lingual root surface and the mesiodistal and buccolingual diameters of the roots were measured by a vernier caliper with a standard error of 0.02 mm (Vernier Caliper Model 93218-0654, Harbin Measuring & Cutting Tool Group Co Ltd., Harbin, PR China). There were no significant differences in the root dimensions among ten subgroups (Table 1).

**Table 1:** Mean dimensions of root lengths and cervical section widths in each subgroup (mm, x ± s, n = 8)

Subgroup	Root length*	Cervical section widths*					
		Mesial	Buccal	Distal	Lingual	M-D	B-L
FP/0	14.91 ± 0.35	2.04 ± 0.24	2.81 ± 0.38	2.06 ± 0.30	2.58 ± 0.22	5.38 ± 0.29	7.88 ± 0.65
FP/1	14.97 ± 0.17	2.24 ± 0.45	2.96 ± 0.47	2.29 ± 0.44	2.85 ± 0.60	5.82 ± 0.95	7.91 ± 0.79
FP/2	14.92 ± 0.18	2.14 ± 0.33	2.81 ± 0.39	2.21 ± 0.30	2.83 ± 0.56	5.70 ± 0.63	8.09 ± 0.40
FP/3	14.89 ± 0.19	2.07 ± 0.26	2.86 ± 0.41	2.14 ± 0.26	2.51 ± 0.24	5.60 ± 0.52	7.87 ± 0.29
FP/4	15.10 ± 0.16	2.07 ± 0.33	2.57 ± 0.31	2.13 ± 0.50	2.42 ± 0.24	5.41 ± 0.46	7.84 ± 0.64
MP/0	14.95 ± 0.30	2.18 ± 0.27	2.93 ± 0.30	2.23 ± 0.25	2.84 ± 0.24	5.69 ± 0.75	8.07 ± 0.49
MP/1	14.88 ± 0.26	2.07 ± 0.34	2.80 ± 0.29	2.27 ± 0.29	2.73 ± 0.41	5.63 ± 0.69	7.96 ± 0.42
MP/2	15.03 ± 0.20	1.96 ± 0.16	2.55 ± 0.25	2.02 ± 0.16	2.44 ± 0.28	5.32 ± 0.28	7.67 ± 0.38
MP/3	15.09 ± 0.24	2.15 ± 0.23	2.89 ± 0.48	2.18 ± 0.23	2.59 ± 0.36	5.61 ± 0.50	7.95 ± 0.71
MP/4	14.96 ± 0.18	2.13 ± 0.16	2.77 ± 0.41	2.25 ± 0.27	2.57 ± 0.33	5.65 ± 0.45	7.87 ± 0.36
* F* Value	0.93	0.60	1.07	0.69	1.56	0.59	0.39
*P* Value	0.50	0.79	0.40	0.72	0.15	0.80	0.94

**Subgroup FP/0**: glass fiber post-core with 0.0 mm ferrule lengths, as control; **Subgroup FP/1**: glass fiber post-core with 1.0 mm ferrule lengths; **Subgroup FP/2**: glass fiber post-core with 2.0 mm ferrule lengths; **Subgroup FP/3**: glass fiber post-core with 3.0 mm ferrule lengths; **Subgroup FP/4**: glass fiber post-core with 4.0 mm ferrule lengths; **Subgroup MP/0**: cast metal post-core with 0.0 mm ferrule lengths, as control; **Subgroup MP/1**: cast metal post-core with 1.0 mm ferrule lengths; **Subgroup MP/2**: cast metal post-core with 2.0 mm ferrule lengths; **Subgroup MP/3**: cast metal post-core with 3.0 mm ferrule lengths; **Subgroup MP/4**: cast metal post-core with 4.0 mm ferrule lengths

*Mean (Standard Deviation); M-D: mesiodistal root width; B-L: buccolingual root width

All the root canals were prepared to 11.0 mm deep for fiber or metal post restoration using No. 2 preshaping drills (#2, 3 M ESPE, St. Paul, MN, USA) with a slow-speed contra-angle handpiece. After root canal preparation, forty roots in FP group received prefabricated glass fiber post-and-core restoration. The post space of the root canal was rinsed thoroughly with an air-water spray and dried lightly with paper points. A resin-based adhesive (Single Bond Universal Adhesive, 3 M ESPE, St. Paul, MN, USA) was applied twice as a thin layer over the walls of the root wall and once over the surfaces of a prefabricated glass fiber post (#2, 3 M ESPE, St. Paul, MN, USA). After thinning lightly with dry, oil-free air, the adhesive was light-cured for 10s at 600 mW/cm^2^ (Variable Intensity Polymerizer Junior, Bisco Dental, Schaumburg, IL, USA) [3]. The post space was filled completely by injecting resin luting cement (RelyX Ultimate Resin Cement, 3 M ESPE, St. Paul, MN, USA), into which the fiber post was inserted. This was followed by light-curing from a coronal direction. A resin composite core with 4.0 mm in height (Filtek Z350XT, 3 M ESPE, St Paul, MN, USA) was built up around the post and light-cured for 40s again. Ferrules with different heights were subsequently prepared in four subgroups. The resin cores with ferrule structure were further milled to a 6°convergence angle by a milling machine (F3/Egro, Degussa AG, Dusseldorf, Germany), and the width of shoulder were kept at 0.8 mm consistently.

**Fig. 2:**
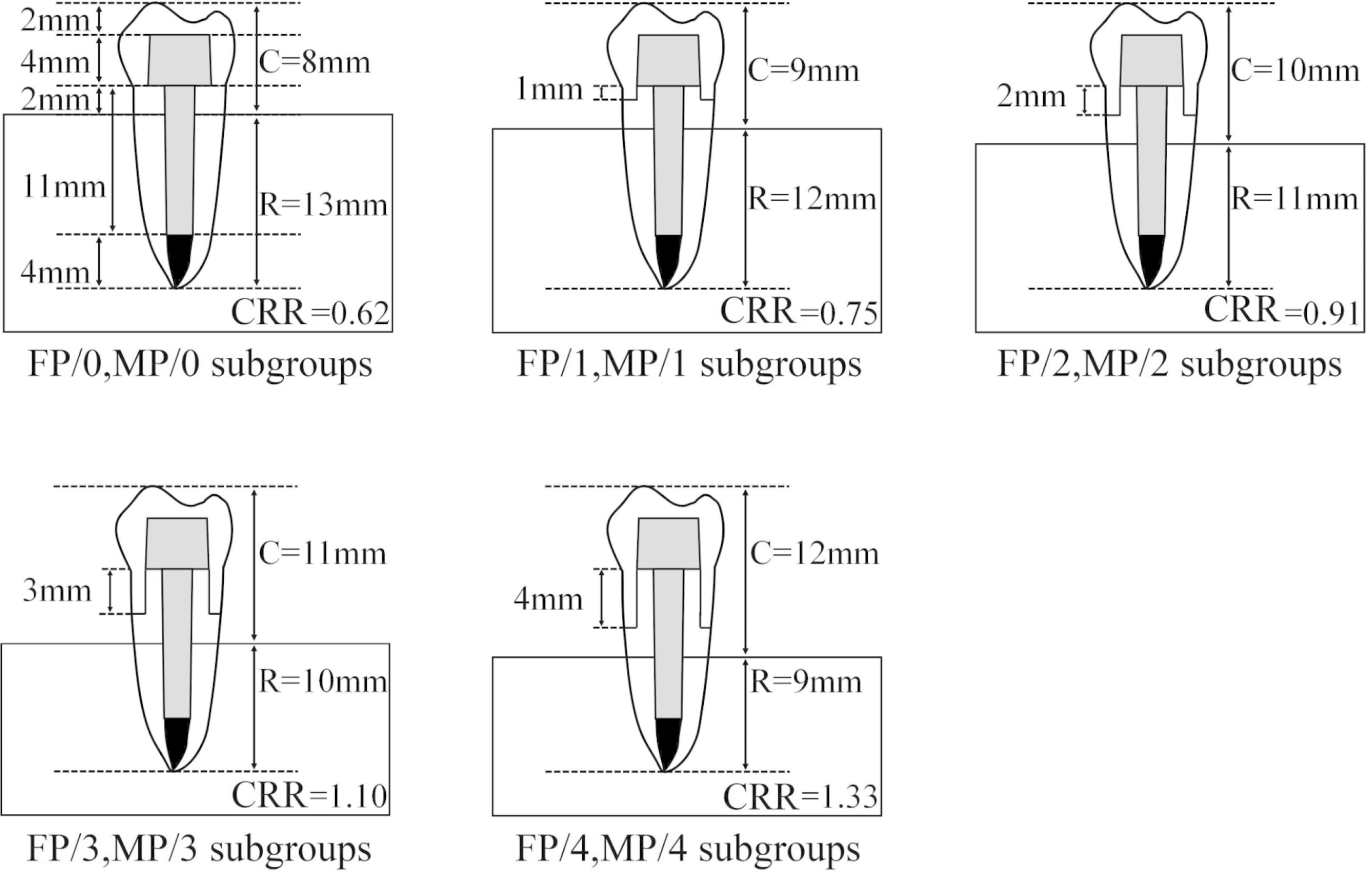
Schematic diagram for the simulated crown lengthening method combined with ferrule designs in each subgroup (C: crown length above bone; R: root length in bone; CRR: crown-to-root ratio)

Another forty roots in group MP were restored using cast metal post-and-cores, the wax patterns of which were prepared first by the same operator with the post of 11.0 mm in depth and the core of 4.0 mm in height. Cobalt-chromium alloy cast metal post-and-cores were then fabricated in dental laboratory and cemented into the post space using glass ionomer cement (Glasionomer, Shofu Inc., Kyoto, Japan). No ferrule was prepared in subgroup MP/0 and 1.0 to 4.0 mm ferrule heights were done in the cervical regions of the roots in subgroups MP/1 to MP/4 respectively. The rest procedures were the same as those in Group FP (Fig. 2).

After storing in 0.9% saline solution for 24 h, standardized Cobalt-Chromium alloy crown with 2.0 mm thickness was made for each tooth in dental lab by CAD-CAM technique. The specimens were prepared by a cementing the crown to the core using glass ionomer cement. The root of each specimen was wrapped with a silicone rubber film (0.2 mm in thickness, Dentsply, Milford, DE, USA) to simulate periodontal ligament and embedded in a self-cured acrylic resin block (Shanghai Dental Materials Manufacture Co., Shanghai, PR China) from 2.0 mm below the cervical margin of the crown. As shown in Fig. 2, the clinical CRRs of the specimens in all subgroups were calculated by taking the cervical embedding plane of the self-cured resin block as the boundary (simulating the crest of alveolar ridge) are 0.62 (subgroups FP/0 and MP/0), 0.75 (subgroups FP/1 and MP/1), 0.91 (subgroups FP/2 and MP/2), 1.10 (subgroups FP/3 and MP/3) and 1.33 (groups FP/4 and MP/4) respectively.

### Fracture resistance testing

**Fig. 3:**
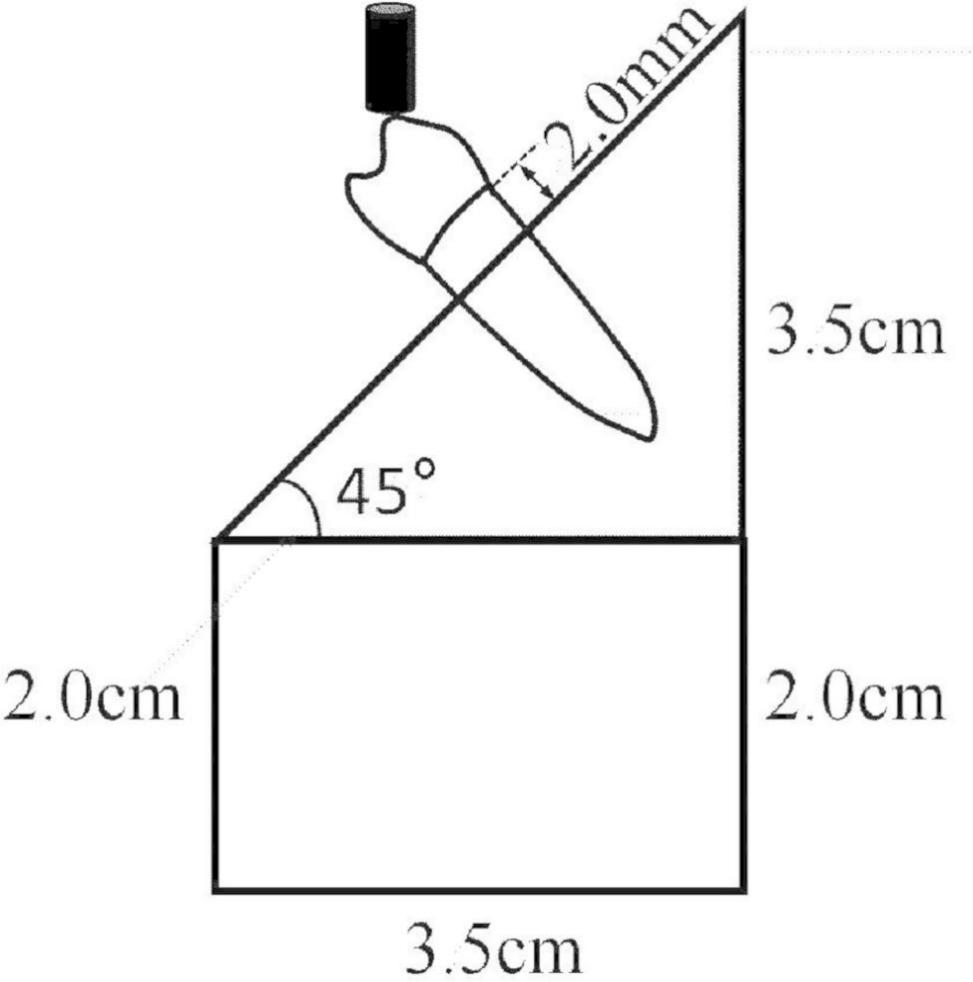
Static mechanical loading

The specimens were loaded on the buccal cusp of the Co-Cr alloy crown at an angle of 135° from the long axis of the root, using MTS810 testing machine (MTS810, MTS Systems Co., Eden Prairie, MN, USA), with the cross-speed of 0.5 mm/minute (Fig. 3). The force (kilonewton, kN) for initial tooth fracture was recorded and the failure pattern noted [3].

### Statistical analysis

SPSS 26.0 software for Windows (SPSS Inc., Chicago, IL, USA) was used for statistical analysis. Data was presented as a mean ± standard deviation for the subgroups. Normality was tested with the Kolmogorov–Smirnov test (*P* > 0.05 was considered for a normal distribution). Two-way ANOVA and Tukey HSD test were used to detect the differences of the fracture strengths between subgroups. The analysis of the regression equation and fitting curve was used to evaluate the effect of internal correlation between CRR and ferrule height on the fracture resistance of residual roots. Fisher’s exact test was used for the difference of the fracture patterns of the specimens. The significant level for statistical analysis was set at α = 0.05.

## Results

**Fig. 4:**
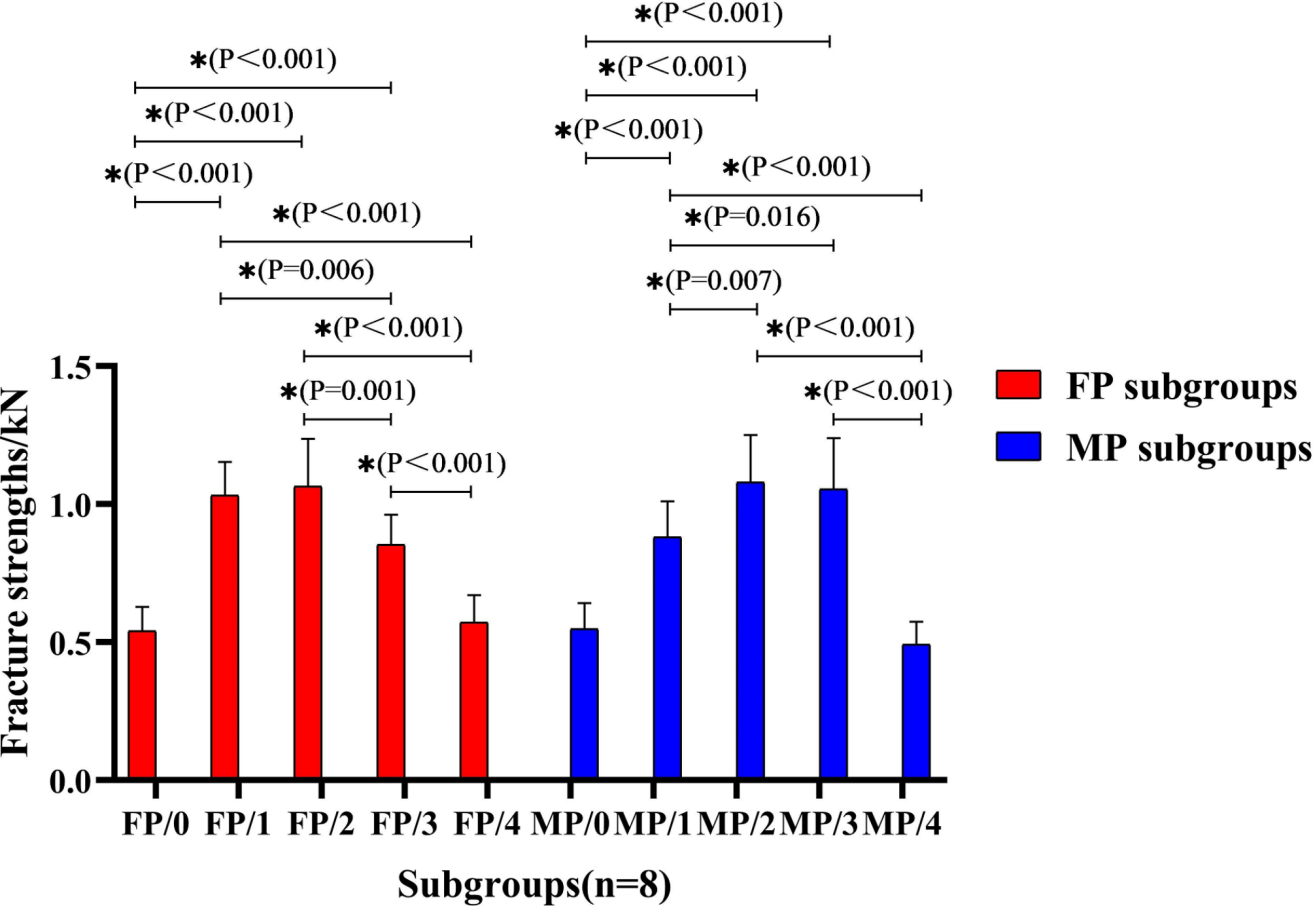
Fracture strengths of the specimens in each subgroup and the statistical analysis between groups (*: significant difference; subgroup codes were defined in Table 1)

**Fig. 5:**
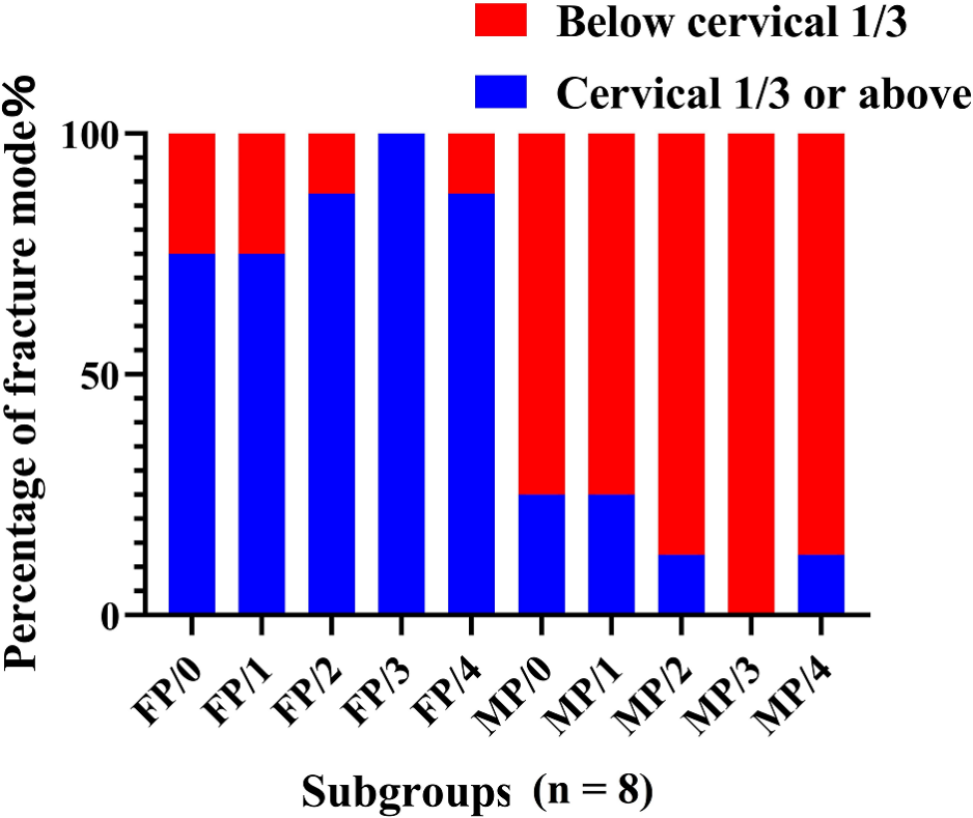
Proportion distribution diagram of fracture patterns in each group

The mean fracture strengths and fracture patterns of the teeth in all subgroups were shown in Figs. 4 and 5. After testing the distribution normality with the Kolmogorov–Smirnov test (P > 0.05), Two-way ANOVA and Tukey HSD test were used for the fracture strengths of the subgroups. The statistical results showed that the factor of ferrule height (or CRR) could significantly affect the fracture resistance of residual roots (F = 60.65, P < 0.001) and no statistical difference was found on the effect of post-and-core material (F = 0.01, P = 0.973). However, there was significant interactions between the two sources of variation (F = 4.14, P = 0.005) (Table 2). Therefore, the null hypothesis that the fracture resistance of endodontically-treated residual roots would not be affected by the ferrule height (or CRR), was rejected; however, that the fracture resistance would not be affected by post material, was accepted.

**Table 2:** Two-way ANOVA table representing effective decomposition of main variables and their interaction

Source	Sum of Squares	df	Mean Square	F value	P value
Ferrule/CRR	4.135	4	1.033	60.652	< 0.001*
Post material	2.000	1	2.000	0.001	0.973
Interaction	0.282	4	0.071	4.142	0.005*
Error	1.193	70	0.017		

*Statistically significant

**Fig. 6:**
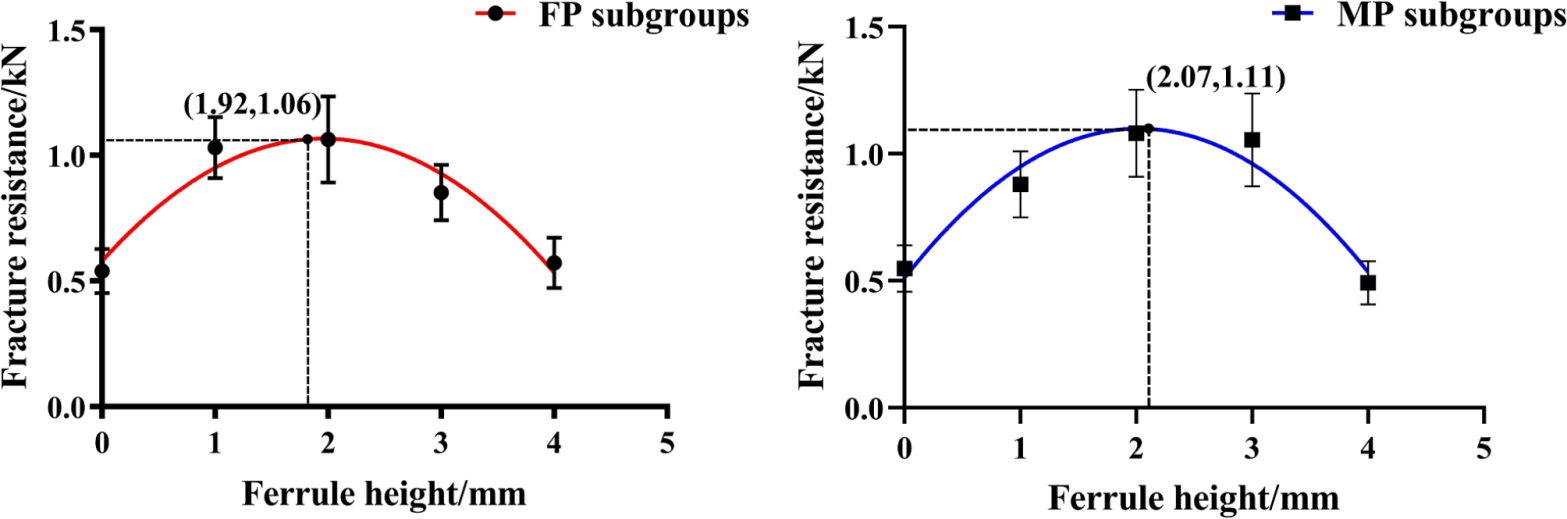
The fitted curve of fracture resistance in FP and MP subgroups(0 ≤ x ≤ 4)

Compared to the control subgroup with no ferrule design in cervical region of the root, a 1.0–2.0 mm ferrule height could significantly increase the fracture strengths of the residual roots, regardless of which post material was used (P < 0.05). If 3.0-mm ferrule height was prepared, the fracture strengths were significantly decreased for the roots with prefabricated fiber post-and-core restoration (P = 0.001), but no statistically difference of the fracture strengths were found in MP group (P = 0.734). Furthermore, the fracture strengths of the residual roots were both significantly decreased in FP/4 and MP/4 subgroups with 4.0 mm ferrule length (P = 0.000) (Fig. 6).

In order to further evaluate the internal relationship between ferrule and CRR in the process of affecting the fracture resistance of the residual roots, the ferrule height was recorded as x and the fracture strengths as y. The analysis of the regression equation in FP and MP groups were obtained as (y = -0.13 × ^2^ + 0.5x + 0.58 (0 ≤ x ≤ 4)) and (y = -0.14 × ^2^ + 0.58x + 0.51 (0 ≤ x ≤ 4)) respectively. The corresponding fitting curves were shown that the highest fracture strength of the specimen restored by prefabricated fiber post-and-core system was found with its ferrule height in 1.92 mm and the matching CRR in 0.90 and that a 2.07 mm ferrule height combined with its CRR in 0.92 would result in the highest fracture strength of the specimen in MP group (Fig. 6). Therefore, the null hypothesis that there was no internal correlation between the ferrule height and CRR on the effect of the fracture resistance of the teeth, was rejected.

In addition, the fracture lines were found mainly at or above the cervical one-third of the roots in FP group, while below the cervical one-third in MP group (Fig. 5). Statistically significant differences in fracture patterns among the groups were found (P < 0.05).

## Discussion

In the restoration of anterior residual crowns or roots, post material [5–9], ferrule design [8, 10–14], CRR [14, 15], the amount of residual tooth tissue [9, 16, 17] and so on are the main factors on the effect of fracture resistance of the teeth. Among them, it has been confirmed by most of the in-vitro studies that a 1.0–2.0 mm ferrule height could significantly increase the fracture strengths of the endodontically-treated residual roots [10, 11]. However, some other studies found that the fracture resistance of the residual roots would be decreased if a certain height of the ferrule was prepared by surgical crown lengthening or orthodontic forced eruption and the clinical crown-to-root ratio was greater than 1 after post-and-crown restoration [3]. However, few studies were focused on the effect of clinical crown-to-root ratio on fracture resistance of the residual roots and crowns, including the internal relationship between ferrule design and clinical crown-to-root ratio on the effect of teeth fracture resistance. For this reason, in this study, a series of ferrule gradients with a height of 0.0 to 4.0 mm were designed in the cervical regions of the residual roots by simulating crown lengthening methods, and CRRs of the specimens after post and crown restorations were evenly distributed from 0.62 to 1.33. The fracture resistance test and the analysis of the regression equation and fitting curve were used to evaluate the effects of ferrule heights, CRRs and their internal relationship on the fracture resistance of the residual roots. During the experiment, sound mandibular first premolars with single canals and similar dimensions (P > 0.05, Table 1), freshly extracted for orthodontic reasons, were used in this study, in order to reduce the selective bias caused by the differences between specimens in each subgroup.

CRR refers to the ratio of the crown to root length of the tooth, including anatomical CRR and clinical CRR. The clinical CRR is calculated by the horizontal line of alveolar bone shown by X-ray film [21]. In this study, CRR specifically refers to the clinical crown-to-root ratio. It is generally believed that the ideal CRR is 1:2, and 1:1.5 is acceptable. The ratio of 1:1 is considered to be the maximum CRR, which can ensure better clinical effectiveness for the restoration of the teeth [22]. In addition, the ferrule effect generated from a certain height of the ferrule preparation in the cervical region of the tooth can resist part of the lateral chewing forces, which helps to improve the fracture resistance of the tooth [12]. The result of this study in Fig. 4 showed that the combination of 1.0-to-2.0-mm ferrule height and CRR within 1 could significantly increase the fracture resistance of the residual roots regardless of the fiber or cast metal post-and-core restoration was used, which further confirmed the positive ferrule effect of the ferrule design.

However, with the increase of the ferrule height by simulated crown lengthening method, the alveolar bone in the cervical area of the residual root was lost and the rotation center of the tooth under forces loading was moved toward the root tip. Accordingly, the height of clinical crown above the fulcrum increased while the root length in bone below the fulcrum decreased, which leaded to the increase of CRR and the decrease of the diameter of the root in the fulcrum level. Therefore, the tooth would tend to be more broken under the action of the lateral forces [23]. Compared to the subgroups with 1.0 and 2.0 mm ferrule height in this study, a 3.0-to-4.0-mm ferrule height prepared by simulated crown lengthening method, reduced the alveolar bone level and the cross-sectional root diameter in the cervical region of the root and increased both the crown length and the CRR values (1.10 and 1.33 respectively), which might lead to the fact that the positive ferrule effect caused by the design of the ferrule was not enough to resist the reduction of the tooth resistance caused by the increase of CRR and the thinning of the root (Figs. 2 and 5). In addition, according to the analysis of the regression equation and fitting curve, the ferrule height and CRR corresponding to the peak of the fracture strengths of the residual roots are 1.92 mm and 0.90 in group FP, and that 2.07 mm and 0.92 respectively in group MP (Fig. 6). Thus, there is a certain correlation between the ferrule design and CRR on the effect of fracture resistance of residual roots. when a certain height of ferrule is prepared by surgical crown lengthening method, the CRR value of the tooth after post-and-core-and-crown restoration should be kept within 0.9 ~ 0.92, no matter what kind of post material is used, so as to facilitate the fracture resistance of the tooth.

Post material had little influence on the clinical efficacy of residual crowns and roots [23], but it was still controversial on the effect of fracture resistance of the teeth [7, 24]. In this study, although the post material had no significant effect on the fracture resistance of residual roots (P = 0.973), there was significant interaction between the post material and the ferrule design (P = 0.005). The high elastic modulus of the metal post increased the bearing capacity and fracture resistance of the tooth, which caused significantly higher fracture resistance of the specimen in subgroup MP/3 than that in subgroup FP/3. Furthermore, because of the differences in elastic modulus between Co-Cr alloy and fiber post, the fracture patterns of the teeth in FP group were significantly different from that in MP group and were more conducive to secondary restoration (Fig. 5), which was consistent with previous studies [12, 24–26].

One of the limitations of the present study is that the specimens were loaded from a single angle and only static loading test was carried out. However, dynamic loading and fatigue cycling has been considered as a reliable method to provide clinically relevant information on the long-term stability, durability and lifetime predictions of restorations [27–30]. Also, the applied teeth used in this study are limited to lower premolars, which may not be generalized to molars since molars usually receive higher stress and different angle of bite force. In addition, effect of different preparation designs such as margin and restoration would also affect the clinical outcome [28]. Therefore, different sites of teeth, with different preparation designs, under dynamic loading or fatigue cycling test would be considered in the future studies.

## Conclusions

Within the limitations of this in vitro study, we suggested that the clinical crown-to-root ratio of the tooth should be kept within 0.90 to 0.92 when a certain ferrule is prepared and cast metal or fiber post system is restored for the residual root, so as to improve the fracture resistance of the tooth. The different fracture patterns of the residual roots suggested that fiber post system is more conductive to secondary restoration when comparing to cast metal post system.

## Data Availability

The data supporting the conclusions of this study is included within the article.

## Abbreviations

CRR: crown-to-root ratio
CEJ: cemento-enamel junction
kN: kilonewton
